# Correction: MCL-1 as a tumor microenvironment-linked survival node in cholangiocarcinoma

**DOI:** 10.3389/fonc.2026.1965020

**Published:** 2026-08-19

**Authors:** Hiroki Hashida, Atsushi Kobayashi, Masatoshi Akagami, Tomohiro Okamoto, Hirokazu Tanaka

**Affiliations:** Department of Gastrointestinal Surgery, Hanwa Memorial Hospital, Osaka, Japan

**Keywords:** apoptosis, chemoresistance, cholangiocarcinoma, desmoplasia, IL-6, leukemia inhibitory factor, MCL-1, tumor microenvironment

The **Data availability statement** is missing from the article. The correct **Data availability statement** is: “The original contributions presented in the article are included in the article, figures, and table. Further inquiries can be directed to the corresponding author.”

The **Ethics statement** is missing from the article. The correct **Ethics statement** is: “This study used a commercially available, de-identified human cholangiocarcinoma tissue microarray. According to the institutional policy of Hanwa Memorial Hospital, separate ethics committee approval was not required. No directly identifiable patient information was accessed.”

The original version of this article has been updated.

